# Optimizing occupancy surveys by maximizing detection probability: application to amphibian monitoring in the Mediterranean region

**DOI:** 10.1002/ece3.1207

**Published:** 2014-08-29

**Authors:** Maud Petitot, Nicolas Manceau, Philippe Geniez, Aurélien Besnard

**Affiliations:** 1 Les Ecologistes de l’Euzière, Domaine de Restinclières34730, Prades-le-Lez, France; 2 Centre d’Ecologie Fonctionnelle et Evolutive (UMR 5175), Ecole Pratique des Hautes Etudes, Biogéographie et Ecologie des Vertébréscampus CNRS, 1919 route de Mende, 34293, Montpellier Cedex 5, France

**Keywords:** Anuran, detection probability, occupancy probability, sampling methodology, species phenology, urodela

## Abstract

Setting up effective conservation strategies requires the precise determination of the targeted species’ distribution area and, if possible, its local abundance. However, detection issues make these objectives complex for most vertebrates. The detection probability is usually <1 and is highly dependent on species phenology and other environmental variables. The aim of this study was to define an optimized survey protocol for the Mediterranean amphibian community, that is, to determine the most favorable periods and the most effective sampling techniques for detecting all species present on a site in a minimum number of field sessions and a minimum amount of prospecting effort. We visited 49 ponds located in the Languedoc region of southern France on four occasions between February and June 2011. Amphibians were detected using three methods: nighttime call count, nighttime visual encounter, and daytime netting. The detection nondetection data obtained was then modeled using site-occupancy models. The detection probability of amphibians sharply differed between species, the survey method used and the date of the survey. These three covariates also interacted. Thus, a minimum of three visits spread over the breeding season, using a combination of all three survey methods, is needed to reach a 95% detection level for all species in the Mediterranean region. *Synthesis and applications*: detection nondetection surveys combined to site occupancy modeling approach are powerful methods that can be used to estimate the detection probability and to determine the prospecting effort necessary to assert that a species is absent from a site.

## Introduction

The distribution and local abundance of a species is generally required to set up conservation strategies. A typical survey protocol consists of several visits to the site, for example, during the breeding season, to make a checklist of encountered species. If no individuals of a given species are found in the studied area, it is tempting to consider that the species is absent. This approach is based on the assumption of perfect species detection (i.e., a detection probability of 1). However, although nondetection of a species may mean the species is truly absent at the site, the species may be present but remain undetected during the survey (Mackenzie and Royle 2005).

In amphibians, a group which is particularly sensitive to global change and thus a good indicator of environmental changes (Stuart et al. 2004), the detection probability is usually less than one and may also be highly dependent on the skill of the observer, the period of the year, the weather and other variables, because of amphibians’ complex phenology and their biphasic activity (MacKenzie et al. 2002, 2003; Schmidt 2003). Many studies on these groups have shown that detectability varies among species, sampling methods, observers, time periods, habitat types, and weather (Bailey Larissa et al. 2004; De Solla et al. 2005; Pellet and Schmidt 2005). Failing to allow for variation in detectability generates unreliable data, especially with respect to false negatives (MacKenzie et al. 2002; Schmidt 2003). In most surveys and monitoring programs, the true distribution of an amphibian species is thus often underestimated (Pellet and Schmidt 2005; Mazerolle et al. 2007; Gomez-Rodriguez et al. 2010a). Yet approaches that estimate detection and take this unreliability into account in animals, such as capture–mark–recapture and distance sampling, are available (Mazerolle et al. 2007). According to Schmidt (2003), these are the only reliable methods of analyzing amphibian demography, population dynamics, and distribution because they explicitly deal with variable detection probabilities that are less than one. As these methods can be time-consuming and expensive to implement, the simpler site-occupancy models developed by MacKenzie et al. (2002) appear to be an adequate alternative for monitoring amphibians (e.g., Schmidt 2003; Gomez-Rodriguez et al. 2010a; Sewell et al. 2010). This method is based on detection nondetection data collected at multiple sites during multiple visits, allowing the probabilities of detection and occupancy to be jointly estimated. The number of sequential nondetections necessary to assert, with a pre-specified confidence, that a species is absent from a site can therefore be calculated using both its detection probability and an estimate of its prevalence in the studied area (Wintle et al. 2012).

Occupancy methods that deal with imperfect detection are not yet widely used among herpetologists, but they are becoming increasingly popular. For instance, Pellet and Schmidt (2005) used them to estimate the regional distribution of four anuran species in Switzerland and to determine the number of visits necessary to infer site absence, based on calling activity. Sewell et al. (2010) also used occupancy methods to optimize a large-scale national survey program (NARRS in Britain), using multiple detection methods. Yet, few studies report the use of these methods in the Mediterranean region (see however Gomez-Rodriguez et al. 2010a; Cayuela et al. 2012), despite the fact that faced with highly unpredictable hydrological conditions, Mediterranean amphibians have evolved various strategies for the onset of breeding (Diaz-Pianiagua 1990; Jakob et al. 2003) that need to be taken into account in designing an efficient survey method. Effective survey design requires a reliable detection probability that can be obtained only if replicates of each method at each visit are included in the protocol.

In order to optimize the survey method used to monitor amphibians from Mediterranean region, our study sought to determine (1) the detection probability of eight amphibian species present in the region, (2) the relative and absolute efficiency of three different detection methods (nighttime call count, nighttime visual encounter and daytime netting) for each species, and (3) the minimum number of surveys required to infer the absence of a species with a certain degree of confidence.

## Materials and Methods

### Study area and data collection

The study area was located in the Languedoc region of southern France, west of Montpellier, between Notre-Dame-de-Londres (Lat. 43°49′N, Long. 3°46′E) to the north and Cournonterral (Lat. 43°33′N, Long. 3°43′E) to the south, and La Boissière (Lat. 43°39′N, Long. 3°38′E) to the west and Prades-le-Lez (Lat. 43°41′N, Long. 3°51′E) to the east. It covered a total of 390 square kilometers. The area has a Mediterranean climate, with dry, hot summers and mild winters, with the maximum rainfall in autumn. The average temperature in the area in 2011 was between 15 and 16°C (1.5°C above the norm) (data from the Hérault Climatological Association: www.ach34.fr). The spring was unusually hot. Rainfall was close to normal (950–1050 mm) over the year. The landscape is characterized by a mosaic of habitats resulting from natural (e.g., fire) and human disturbance (e.g., agriculture, grazing). However, an abrupt discontinuation of pastoral activity and the abandonment of agricultural land during the 20th century have led to a rapid increase in the recovery of wasteland by shrubland, followed by woodland (Debussche et al. 1999).

We selected 49 temporary ponds in the study area in order to represent a diversity of typologies (in size, depth, and vegetation coverage). At the beginning of our study, the average area of the ponds was 180 m² (ranging from 23 to 459 m^2^). The depth ranged from 30 cm to over 1 m (depth over 1 m could not be measured). The ponds were diverse in terms of vegetation coverage (mean 40%, ranging from 0% to over 75%), sun exposure (mean 60%, ranging from 25% to 100% of wetland surface exposed to direct sunlight) and pond-bottom type (artificial or natural). The main amphibian breeding habitat in this area are man-made ponds often dug out to provide drinking water for livestock (sheep and goats).

All 49 sites were visited by the same observer (MP, first author) four times during the breeding season, (25 February–15 June) to maximize the chance of detecting the ten species known to be present in this area (see below). The observer was experimented and familiar with the Mediterranean region and especially with the studied area. Three detection methods were systematically used at each visit to each pond: (1) ***nighttime call count*** (anurans only), with three equidistant listening points and a two-minute break between each listening point, (2) ***nighttime visual encounter*** (anurans and urodelans) using a Xanlite torch (beam range of 200 m) while walking pond shores along 3 m transects, separated from each other by 3 m (mean 6, ranging from 3 to 13), (3) ***daytime netting*** (anurans and urodelans) using a standard dip net (4-mm mesh). The number of dip net sweeps was proportional to the surface area of each pond (mean 9, ranging from 3 to 18) and was divided among different microhabitat types within the pond: aquatic vegetation and open water. The distance between dip net sweeps was standardized to 3 m. Night time searches and acoustic surveys allow the detection of adults while dipnet survey allows the detection of both larvae and adults. As the density of larvae increases during the breeding period, the efficiency of this method should increase during the season.

Captured animals (adults or larvae) were immediately identified and released. All the equipment was disinfected with 70° alcohol between each site in order to reduce the risk of disease transmission such as chytridiomycosis. The detection or nondetection of each species was separately recorded for each method and each sampling unit (for each listening point, shore transect, and dip-net sweep). Using one experienced person to detect amphibians reduced the chance of falsely detecting species. Moreover, all pond sites were separated from each other by at least 500 m and were visited every 3 or 4 weeks in a random order. The following parameters were recorded at each visit: water depth (ruler of 1.50 m), surface area (laser rangefinder; Bosch Leinfelden-Echterdingen, Germany), water temperature (pH and EC combination tester, Hanna), air temperature (thermo-hygrometer, Pierron), and vegetation coverage (Braun-Blanquet 1932).

We detected all 10 species known in the studied area: 8 anurans (*Alytes obstetricans*, *Bufo bufo*, *Bufo calamita*, *Discoglossus pictus*, *Hyla meridionalis*, *Pelobates cultripes*, *Pelodytes punctatus*, *Pelophylax* sp.) and 2 urodelans (*Lissotriton helveticus* and *Triturus marmoratus*). The various green frog species (*Pelophylax ridibundus, P. kl. grafi,* and *P. perezi*) could not be separately identified, and were therefore all referenced as *Pelophylax* sp. They form a group of species that are very difficult to distinguish due to hybridizations (Crochet et al. 1995; Pagano et al. 2001). *B. bufo*. and *B. calamita* larvae are also difficult to distinguish from each other in the early stages. In order to avoid misidentification, larvae of these two species were identified only after the hind legs developed (at which time a distinctive white spot appears on the throat of *B. calamita* larvae) (Miaud and Muratet 2004).

### Data analysis

All analyses were conducted separately for each species, using the unmarked library (Fiske and Chandler 2010) in R 2.11.1 statistical software (R core team 2009). We used single-season occupancy models developed by MacKenzie et al. (2002) to estimate both the detection probability (*P*) and the proportion of occupied sites (*ψ*). The detection histories were built using all the methods and their replicates and were thus 36–136 long: four visits with three different methods replicated a certain number of times (see materiel). This model assumes population “closure”, that is, that site occupancy is constant throughout the survey. We considered in our study that the population was closed because all species have been detected by one of the three methods at least once during the first and the last visit. Thus, species are all present in the vicinity of the ponds. Yet, they may remain undetected at certain places because they were not in the immediate vicinity of the ponds and thus were unavailable for detection. However, availability is a part of the detection process we are interested in. In this study, we fitted a temporal covariate (date or quadratic date effect, see below) that allowed capture the availability process. Moreover, Schmidt (2005) showed that in pond-breeding amphibians, a small departure from closure assumption did not affect his results. However, the absence of a species in a given month at a given pond could be due either to the species being missed by the observer when it was in fact present (undetected while available for detection) or to its true absence in or around the pond because breeding had not yet started or was already finished (unavailable for detection). To acknowledge this potential issue, recent studies of amphibian pond occupancy have used a monthly survey design assuming that modeling the monthly probability of detection better allows the availability for detection to be estimated (Gomez-Rodriguez et al. 2010b; Gomez-Rodriguez et al. 2012). Such a design may, however, provide biased detection estimates since it models relative monthly detection probabilities and not absolute detection probability for each month. The same holds true if several methods are used simultaneously. One way to deal with such limitations is to design a survey based on the replication of the sampling unit at each visit for each survey method, as we did in this study. This allows absolute detection to be estimated for each survey method or month.

We developed three detection probability models. In the first model, we assumed that both site occupancy and detection probability were constant. In the second model, we included the date of the survey and the method of detection plus the interaction between date and method in order to account for species phenology and variation in method effectiveness regarding this phenology. Both linear and quadratic relationships of date and detection probability were tested, as previously done by Pellet and Schmidt (2005). Quadratic effects of the date were tested in order to detect a potential peak in amphibian breeding activity. In the third model, we assumed that site occupancy was constant, but that detection probability was affected by the method of detection and by water temperature. In this model, the survey date was replaced by water temperature in order to determine whether the date is a good proxy for the effects of abiotic factors impacting amphibian phenology. The correlation between date and water temperature was strong (*r* = 0.75), so these two covariates could not be tested simultaneously in the model. We then tried to determine which one better explained the detection probability of the species.

Akaike’s Information Criterion (AIC) was used to rank models (Burnham and Anderson 2002). It is calculated by AIC = Deviance + 2*np (with np being the number of parameters). This criterion represents a compromise between a good fit of the model to the data and a limited number of parameters (parsimony). The optimal fitted model is identified by the minimum AIC value and models are considered as competitive when ΔAIC is superior to 2 (Burnham and Anderson 2002). Models with a delta AIC<2 were considered as equivalent in this study. All models were fitted using the unmarked library (Fiske and Chandler 2010) in R 2.11.1 statistical software (R core team 2009).

According to Wintle et al. (2012), the number of visits necessary to ascertain that a species is truly absent from a site with a certain degree of confidence can be calculated if a prior knowledge of detection probability and of occupancy probability (prevalence in the area) is available. If p represents the probability of detecting a species (assuming it is present at a site) and *γ* the probability that the species is present in the sites, then the probability *α* of not seeing a species after *N* visits is [log(*α*/(1–*α*))-log(*γ*/(1–*γ*))]/log(1–*p*). Here we obtained the *γ* through a regional database that compiles more than 50,000 observations of amphibians from 1966 to 2011 in the Languedoc-Roussillon region (Geniez and Cheylan 2012). For each species, we calculated for the 122 known ponds in the study area, the proportion of ponds for which at least one observation of the target species has been reported over the last 10 years. Yet, since prevalence may be difficult to estimate even with our data base, we also used the classical method to estimate that number of visits (see e.g., Pellet and Schmidt 2005). We also used in these formulae the detection probability (*p*) we estimated through our site occupancy models. The detection probability was separately calculated for one survey method (nighttime call count), two combined methods (nighttime call count and nighttime visual encounter) and three combined methods (nighttime call count, nighttime visual encounter and daytime netting). We considered a standard visit to consist of 2 listening points, 6 transects, and 9 dip-net sweeps. The number of replications of each method at a site is purely subjective; we chose a number close to what could be easily set up in the field. However, the detection probability of a species can be easily calculated for any type of effort. For example, for our defined standard visit (2 listening points, 6 transects, and 9 dip-net sweeps), the probability of detecting at least one species with a detection probability P_calling_, P_searching_, and P_netting_ is:

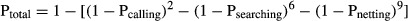


Our results indicated the minimum number of visits necessary to be 95% certain (*α* = 0.05) that a species is absent from a surveyed site.

## Results

### Pond occupancy

During the study, we detected the ten species known to be present in the study area. However, sufficient data could be obtained for eight species only. For these eight species, the estimated site occupancy is close to the naïve estimation (Table 1), suggesting that our labor-intensive survey design performed well in detecting species at the site they occupy.

**Table 1:** Models fitted and estimation of site occupancy for the eight species detected in 49 ponds of southern France. Naïve *ψ*: the naïve occupancy estimate corresponds to the number of ponds where the species was detected divided by the total number of ponds. Est. *ψ*: the estimated occupancy corresponds to the proportion of ponds occupied by the species, as estimated by the best fitted model

Species	Naïve *ψ*	Est. *ψ*	Models	AIC	∆ AIC
*Alytes obstetricans*	0.163	0.167 ± 0.05	p(.), *ψ*(.)	385.82	
			p(method*date), *ψ*(.)	337.75	−48.07
*Bufo calamita*	0.163	0.188 ± 0.05	p(.), *ψ*(.)	343.45	
			p(method*date), *ψ*(.)	339.01	−4.44
			p(method*T_water_+T²_water_), *ψ*(.)	314.78	−28.67
*Bufo bufo*	0.367	0.438 ± 0.07	p(.), *ψ*(.)	1375.39	
			p(method*date), *ψ*(.)	1195.60	−179.79
			p(method*T_water_+T²_water_), *ψ*(.)	1089.37	−286.02
*Pelophylax* sp.	0.673	0.707 ± 0.06	p(.), *ψ*(.)	2655.58	
			p(method*date), *ψ*(.)	2014.51	−641.07
			p(method*T_water_+T²_water_), *ψ*(.)	1942.92	−712.66
*Pelodytes punctatus*	0.632	0.632 ± 0.06	p(.), *ψ*(.)	1834.36	
			p(method*date), *ψ*(.)	1323.2	−511.16
			p(method*date+date²), *ψ*(.)	1281.07	−553.29
*Hyla meridionalis*	0.918	0.959 ± 0.03	p(.), *ψ*(.)	3781.98	
			p(method*date), *ψ*(.)	3132.71	−649.27
			p(method*T_water_+T²_water_), *ψ*(.)	2992.50	−789.48
*Triturus marmoratus*	0.612	0.619 ± 0.07	p (.), *ψ*(.)	1484.03	
			p(method*date), *ψ*(.)	1365.91	−118.12
			p (method*date+date²), *ψ*(.)	1358.82	−125.21
*Lissotriton helveticus*	0.938	0.938 ± 0.03	p(.), *ψ*(.)	3091.34	
			p(method*date), *ψ*(.)	2893.7	−197.64
			p (method*date+date²), *ψ*(.)	2868.30	−223.04

*Hyla meridionalis* and *L. helveticus* are ubiquitous species since the respective estimates suggest they occupy 45 and 46 of 49 sites, that is, more than 90% of the ponds. *T. marmoratus*, *P. punctatus,* and *Pelophylax* sp. were found in 30, 31, and 33 of the 49 sites respectively, i.e., more than 60% of the ponds. *B. bufo* occupies 18 of 49 sites, that is, 37% of the ponds. *B. calamita* and *A. obstetricans* were found in only 8 of 49 sites, that is, 16% of the ponds. Unfortunately, the data set for *Pelobates cultripes* and *Discoglossus pictus* was too small to perform an occupancy analysis. They were present in only 3 of 49 sites and were rarely detected. *P. cultripes* was never heard in a call survey, and was observed on only three occasions: 10, 22, and 23 March. Its larvae were captured on 18 May and 9 June. *D. pictus* was detected only by nighttime visual encounter on four occasions: 9 and 11 March and 4 and 16 May.

### Estimated detection probability using the detection method and survey date as covariates

For all species, the models that included an effect on the detection probability of the date and the survey method in interaction were systematically better than the null model (Table 1).

**Figure 1:**
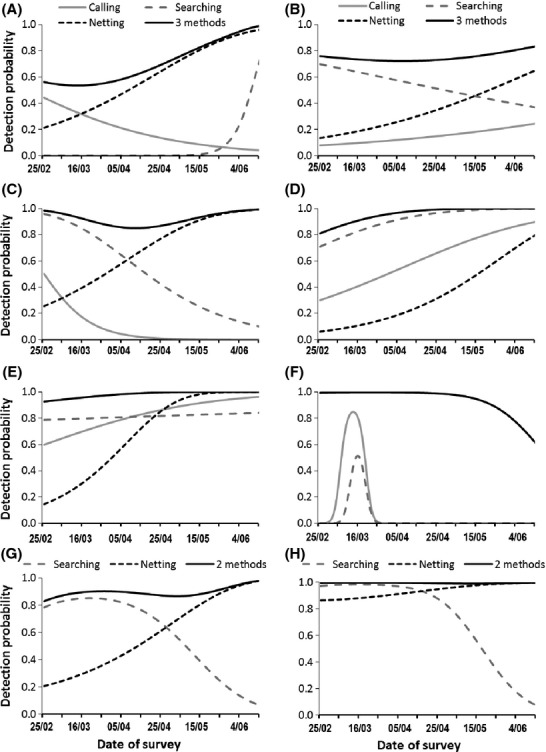
Estimated detection probability of the different survey methods: Calling (nighttime call count using two listening points), Searching (nighttime visual encounter using six transects), and Netting (daytime netting using nine dip nets). Detection probability was estimated using site-occupancy modeling on eight species of amphibians detected in 49 ponds of southern France: (A) *Alytes obstetricans*, (B) *Bufo calamita*, (C) *Bufo bufo*, (D) *Pelophylax* sp., (E) *Hyla meridionalis*, (F) *Pelodytes punctatus*, (G) *Triturus marmoratus*, (H) *Lissotriton helveticus*.

**Figure 2:**
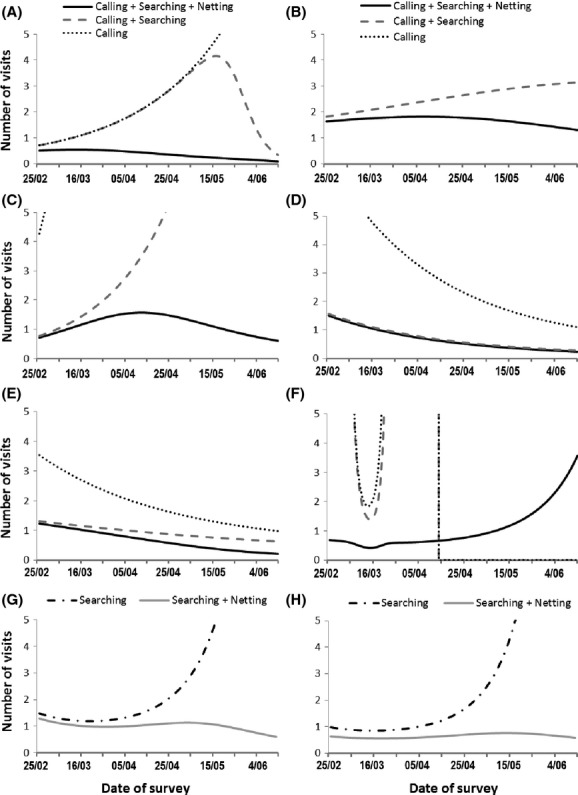
Number of visits required to detect amphibian species using one, two, or three methods of detection estimated by using site-occupancy modeling on eight species of amphibians detected in 49 ponds of southern France: (A) *Alytes obstetricans*, (B) *Bufo calamita*, (C) *Bufo bufo*, (D) *Pelophylax* sp., (E) *Hyla meridionalis*, (F) *Pelodytes punctatus*, (G) *Triturus marmoratus*, (H) *Lissotriton helveticus*.

The best fitting models, with the date as a covariate in detection probability, include either a linear or quadratic relationship, depending on the species and the method (Table 1). Figure 1 presents the detection probability obtained using nighttime call count (calling), nighttime visual encounter (searching), or daytime netting (netting) and the three methods all together. The mean detection probability varies greatly between species, as does the effectiveness of each method and the trend in their effectiveness with the date. No clear common pattern could be identified between the different species. The use of one, two or even three methods is often required to achieve a detection probability over 95%, and consequently to allow the number of visits to be reduced. This conclusion is the same whether we used the equation based on the prevalence (Fig. 2) or the classical equation (see Appendix S1). As it can be seen from Fig. 1 and Table 2 for all species but the *Bufo calamita*, the optimal detection probability at a specific date combining the three methods always reach 1 meaning that if the date is well chosen and if the three detection methods are used most of the species are detected in a single visit.

**Table 2:** Maximized detection probability (*p*) and number of surveys required, using the three combined methods

Species	*Optimal detection probability* (95% confidence)	No. surveys needed (95% confidence)
*Alytes obstetricans*	0.99 [0.82–1.00]	0.09 [0.05–0.24]
*Bufo calamita*	0.83 [0.55–0.95]	1.29 [0.76–2.88]
*Bufo bufo*	0.99 [0.98–1.00]	0.59 [0.47–0.79]
*Pelophylax* sp.	1.00 [1.00–1.00]	0.23 [0.20–0.27]
*Pelodytes punctatus*	0.99 [0.97–1.00]	0.68 [0.53–0.95]
*Hyla meridionalis*	1.00 [1.00–1.00]	0.21 [0.19–0.23]
*Triturus marmoratus*	0.98 [0.91–1.00]	0.57 [0.41–0.92]
*Lissotriton helveticus*	1.00 [1.00–1.00]	0.57 [0.46–0.75]

For *H. meridionalis* and *Pelophylax* sp*.,* nighttime visual encounter surveys were an efficient method: a high detection probability (*P* > 0.8) was achieved from early March to mid-June for a single visit. Nighttime call count was also useful from April to June for *H. meridionalis*, and from around mid-May to June for *Pelophylax* sp. The combination of nighttime call count and visual encounter provided a detection probability of 1 for a single visit from mid-March to June. Consequently, only one visit would be necessary to be 95% certain of the absence of the species if these two methods were used between mid-March and June. For these two species, netting does not provide further information (Fig. 2).

In contrast, for *A. obstetricans*, a low detection probability was obtained using nighttime call count on a single visit (*P* ≤ 0.4) or nighttime visual encounter (*P* = 0) from the end of February to the end of May. A sharp increase in detection probability obtained by nighttime visual encounter occurred in early June. However, this increase was due to the visual observation of only one adult on two different ponds. This species is very difficult to observe, so this result should be treated with caution. Netting was much more effective, and is therefore required to achieve a detection probability higher than 0.6 on a single visit. Netting should be performed between late April and mid-June to be optimal (Fig. 1). If the three methods are combined, only one visit conducted between February and June is needed to be 95% sure that a site with no detection is unoccupied (Fig. 2).

Nighttime visual encounter provided a higher detection probability than call count for *B. calamita* (Fig. 1). A relatively high detection probability (0.5 ≤ *P* ≤ 0.7) can however be reached in a single visit by combining these two methods. The addition of netting ensures a constant and higher detection probability whatever the period (0.7 ≤ *P* ≤ 0.8). Therefore, a limited number of two visits are required between March and June to be 95% sure of detection if the three methods are combined (Fig. 2).

For *B. bufo,* nighttime visual encounter also provided higher detection probability than call count over the entire studied period (Fig. 1). However, after April, the detection probability obtained by visual encounter decreased, and netting was required to reach a good level of detection. One to two visits between March and June are necessary to detect this species if the three methods are combined, while more than four visits are required between April and June if netting is not used (Fig. 2).

*Pelodytes punctatus* presents a very distinctive profile compared to the other species. Its detection peak obtained by visual encounter or call count is very short. The species can therefore be easily missed, since the pond must be visited during that very short period. However, netting was very effective, especially from March to mid-May, and is therefore required to ensure a good level of detection for this species. If netting is used, only one visit before the end of May is needed (Fig. 2).

For *T. marmoratus* and *L. helveticus,* visual encounter provided a high detection probability (*P* ≥ 0.8) for a single visit performed between the end of February and the beginning of April. After that period, the use of netting was necessary to maintain a high detection probability (Fig. 1). One to two visits between March and June are necessary to detect these two species with a 95% degree of confidence if both methods are combined (Fig. 2), while more than three visits are required between the end of April and June if netting is not used.

### Estimated detection probability using detection method and water temperature as covariates

The model including the temperature as a covariate on detection was better than the model including the sampling date for *B. bufo*, *B. calamita*, *Pelophylax* sp., and *H. meridionalis* (Table 1). For these species, temperature was a better predictor of detection probability than date.

**Figure 3:**
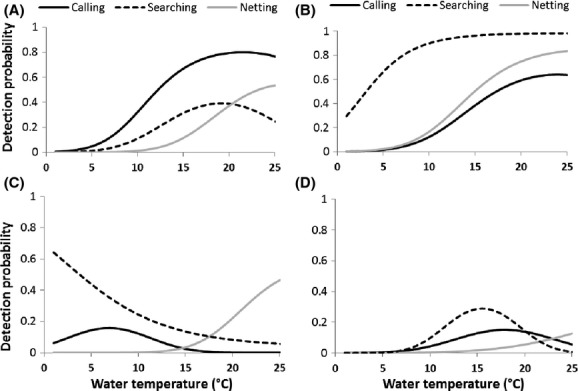
Relationship between water temperature and detection probability for (A) *Hyla meridionalis*, (B) *Pelophylax* sp., (C) *Bufo bufo*, and (D) *Bufo calamita*.

The optimal water temperature for nighttime visual encounter or call count varied among species (Figure 3). *H. meridionalis* was more often detected (by call count and visual encounter) when the water temperature was higher than 19°C. For *Pelophylax* sp., a water temperature above 10°C was required to detect this species visually, and above 20°C to detect it by call. The impact of water temperature on detection probability of *B. bufo* and *B. calamita* was less pronounced. Very low water temperature was required to detect *B. bufo* by call or visually, whereas *B. calamita* was more often detected when water temperature was between 15°C and 20°C.

## Discussion

Our results show that detection probability varies among species, sampling dates, and method used. They also indicate that detection is close to 1 for all species in a single visit if the date is well chosen. Since the peak of detection probability is not the same for all species, three visits regularly spread out between mid-March and the end of May combining nighttime call count, nighttime visual encounter, and daytime netting should allow detecting all the species. Note that the observer was familiar with the amphibians from Mediterranean region and especially with the studied area. This might have positively affected detection probability compared to what could have been obtained by a novice. Yet, the study of amphibians and especially their identification on larvae needs high level of expertise. Such studies are thus usually performed by local experienced observers that might have the same level of expertise we had.

### Comparison of detection methods

Not surprisingly, *H. meridionalis* and *Pelophylax* sp. were relatively easy to detect by call. *P. punctatus* was also easy to detect by call, but only during a very short period of time. Its detection peak occurred in March, in line with the results from a recent work by Geniez and Cheylan (2012). For all other anuran species, *A. obstetricans*, *B. bufo*, and *B. calamita*, call count was not effective enough to be 95% sure of detection with a moderate effort. Although call surveys are widely used to monitor both distribution and abundance (Crouch and Paton 2001; Pierce and Gutzwiller 2007), they cannot be systematically used to detect all anuran species. Pellet and Schmidt (2005) demonstrated that more effort is necessary to detect *B. calamita* by call (*P* = 0.442, number of visits = 6) than *H. arborea* (*P* = 0.742, number of visits = 3) in Switzerland, similarly to what we observed in our study. They also found a relatively higher detection probability for *A. obstetricans* (*P* = 0.570 vs. *P* < 0.1 in our study) but they suggest a careful interpretation of these results since the species was detected at only 3 of 27 sites. Detectability problems can occur for species with a very brief calling period or species that tend not to form dense choruses, but rather call sporadically or at relatively low volume (De Solla et al. 2005). For example, *B. calamita* and *H. meridionalis* have very loud calls that can be heard more than 1 kilometer away in good conditions, whereas *A. obstetricans* has a soft, high call that can be confused with that of the Scops owl (Pellet and Schmidt 2005). According to our observations, *H. meridionalis* frequently formed a dense chorus that could have masked the calls of quieter species such as *A. obstetricans*, for example. Moreover, the size of the population may affect calling. For *B. calamita*, the decision to call depends on density: sometimes the males of this species call to attract females, while other times they behave as silent satellites (Arak 1988). The relationship between population size and calling may impact detectability, so it is possible that low density populations could be missed even if several visits are performed. Generally speaking, the abundance of a species directly impacts its detection probability: the more abundant, the easier to detect (MacKenzie et al. 2006; Tanadini and Schmidt 2011).

Nighttime visual encounter is well adapted for the detection of almost all studied species (*H. meridionalis*, *Pelophylax* sp., *B. bufo*., *B. calamita*, *T. marmoratus*, *L. helveticus*), with the exception of *A. obstetricans* and *P. punctatus*, which were rarely detected using this method. Visual encounter surveys are easy to implement and only moderately disturb most studied species. High levels of water turbidity and inaccessible pond edges can, however, make this method less effective, reducing its detection probability (*personal observation*). Moreover, nighttime visual observation may result in misidentification of some species, especially if the survey is carried out by volunteers.

Although most of the species in our study could be detected with a great level of confidence by combining nighttime call count and visual encounter only, a high detection probability of *A. obstetricans* and *P. punctatus* required netting. Daytime netting is also recommended for detecting *L. helveticus*. Contrary to what was observed for all other studied species, adults of *L. helveticus* were frequently captured by netting, especially early in the season. Therefore, a high detection probability can be obtained using searching and/or netting, as already demonstrated by Sewell et al. (2010) who obtained high detection probability using daytime encounter, night counts, netting but also funnel trapping (0.63 < *P* < 0.81 vs. *P* = 0.99 in our study). For all other species, adults were rarely captured. Unfortunately, no comparable result can be found in the literature as dip netting is mainly used for larval sampling, and the detection of adults with this technique is excluded (Gomez-Rodriguez et al. 2010a), or different methods are combined (e.g., dip netting and visual encounter) and the detailed results obtained for each separate method are not provided (e.g., Sewell et al. 2010).

It should be noted that, according to Sewell et al. (2010), newt species (*L. helveticus*, *L. vulgaris,* and *Triturus cristatus*) are under-recorded by combining netting, nighttime visual surveys, and nighttime call count surveys. They recommend incorporating the additional method of bottle-trapping into survey methodologies wherever possible. However, the exclusion of larvae and females from their counts (as female and larvae of *Lissotriton vulgaris* can be easily confused with females or larvae of *L. helveticus*) may explain the low detection probability they obtained by visual encounter and netting. In our studied area, larvae and females of newt species *T. marmoratus* and *L. helveticus* are easily distinguishable and were therefore included in our protocol. Our results showed that netting is well adapted for detecting both species. For all anuran species (except *H. meridionalis* and *Pelophylax* sp.), the combination of netting with call and/or visual surveys reduced the number of visits needed to the site to detect them. Netting also has the advantage of verifying that the pond is used for breeding. On the other hand, disadvantages of netting include the risk of disturbing the species, as well as the need to disinfect nets between sites to minimize the risk of spreading disease. It also requires a high level of experience in larvae identification, especially in early larval stages. Thus, the effectiveness of the different detection methods depends on the species considered. Moreover, as the breeding period of amphibians is limited in length, effectiveness also depends on the date of the survey.

Whatever the survey method used, the experience of the observer can also be an important factor in sampling variation and bias in the detection of low density populations (Fitzpatrick et al. 2009). Training can reduce interobserver variability.

### Species phenology and detection probability

Amphibian phenology varied greatly among species. Both the breeding period (early or late breeders) and the length of adult detection on breeding sites (and the length of larval development) differs between species. *B. bufo*, *P. punctatus*, *T. marmoratus,* and *L. helveticus* adults were more often detected early in the season (February to April). In contrast, *H. meridionalis* and *Pelophylax* sp. adults were more often detected late in the season (April to June). These results are in line with the results found by Geniez and Cheylan (2012). *A*. *obstetricans* and *B. calamita* had intermediate behavior. *B. calamita* was easier to detect by nighttime visual encounter early in the season (March) and easier to detect by nighttime calling late in the season (June). However, *A. obstetricans* was more often heard early in the season (February) and more often seen late in the season (June). According to Geniez and Cheylan (2012), the peak detection of adults occurs in April for *B. calamita* and in May for *A. obstetricans*. The higher detectability of *A. obstetricans* obtained by call surveys early in the season in our study is therefore surprising. Its relatively quiet call, supplanted by the call of *Hyla meridionalis* late in the season, and the small amount of data obtained for this species in our study may explain this result.

Most species were easy to detect by netting from April, except for *P. punctatus* larvae, which were detected at the beginning of the study (February). In Mediterranean populations, *P. punctatus* reproduces in spring, but also in autumn (Jakob et al. 2003; Jourdan-Pineau et al. 2012; Geniez and Cheylan 2012). Its autumn tadpole can survive the winter and can therefore be detected by netting earlier in the season. *P. cultripes* (and anecdotally *B. calamita* and *H. meridionalis*) can also have a bimodal breeding strategy in the Mediterranean region, but the highest reproductive effort occurs in spring (Jakob et al. 2003; Richter-Boix et al. 2006; Geniez and Cheylan 2012). In our study, no larvae of these species were observed early in the season, suggesting that no breeding occurred the previous autumn.

However, the reproductive strategy of amphibians can vary in different years in Mediterranean ponds. Temporary Mediterranean ponds are characterized by the unpredictable date of annual pond flooding and drought (Jakob et al. 2003). Strategies to cope with this unpredictability include plasticity in the onset of breeding. For instance, although both newt species (*L. helveticus* and *T. marmoratus*) and *B. bufo* have narrow timeframe of reproduction, *H. meridionalis*, *P. punctatus*, *Pelophylax perezi, B. calamita,* and *Pelobates cultripes* are more or less plastic in terms of their reproductive period, depending on when annual precipitation and therefore pond flooding occurs (Jakob et al. 2003; Richter-Boix et al. 2006). As a result, an absolute date may not be the best indicator to use for planning surveys.

Knowledge of the factors that affect phenology and, in turn, detection probability can therefore help to optimize monitoring programs. For example, the relationship between temperature and detection probability we obtained for four species could be used to determine ideal conditions for anuran surveys, as also demonstrated by Pellet and Schmidt (2005) and Sewell et al. (2010). However, as we have demonstrated, optimal conditions may vary greatly among species, and these conditions are not always well known. More work is needed to describe in detail the relationship between climatic covariates (such as ambient temperature or rains) and amphibian phenology before climatic covariates could be used to plan field sessions. Moreover, relying on climatic covariates requires that field workers be flexible enough to adapt their efforts specifically to weather conditions, which may not be realistic. As our study demonstrates, a three-visit protocol ensures a high level of detection for all species when these visits are spread over the entire breeding season and the three survey methods are combined. Coordinating the survey with weather conditions is thus not especially useful unless the goal is to estimate abundance or to set up capture–recapture protocols, for instance.

## Recommendation for Survey Protocols

Our results indicate that if the objective was to detect all species present at a Mediterranean pond in a limited number of visits, it is advisable to combine three detection methods (nighttime call count, nighttime visual encounter, and daytime dip-netting) and to perform a minimum of three successive visits: one between mid-March and the beginning of April, one between mid-April and the beginning of May, and one at the end of May. This protocol ensures a detection probability of over 95% for all species. However, if possible, call and visual surveys should be more intensively used, as dip netting can be much more disturbing and should be used with caution. Dip netting should be conducted with disinfected equipment (to minimize the risk of disease development), during the day (when most adults are out of the pond) and very delicately (to minimize the risk of destroying spawning sites). In terms of timing the field sessions, as Mediterranean ponds are characterized by unpredictable hydrological conditions, the date of the site visits should be adapted to the meteorological conditions, in terms of when flooding creates temporary ponds. This study was carried out during a single breeding season, so further work would be necessary to estimate variation in detection probability in different years and to determine causal factors. However, our study obtained similar results on species phenology to the work of Geniez and Cheylan (2012), resulting from the compilation of some 50,000 observations of amphibians from 1966 to 2011 in the Languedoc-Roussillon region.

The optimization of survey protocols for biodiversity monitoring is crucial in a context of financial limitations. This is especially true in declining species for which monitoring drives money that may be better used for actions (Cleary 2006). Here, we demonstrated, on a group that may be difficult to monitor because of its phenology, that a pilot study based on detection non-detection data coupled with site occupancy modeling approach are especially efficient for such an optimization. If possible, we highly recommend building site occupancy design.

## Supporting Information

Appendix S1 Number of visits required to detect amphibian species by using one, two, or three methods of detection estimated by using occupancy modeling approach on the 8 species of amphibians detected in 49 ponds of southern France. (A): *Alytes obstetricans*, (B): *Bufo calamita*, (C): *Bufo bufo*, (D): *Pelophylax* sp., (E): *Hyla meridionalis*, (F): *Pelodytes punctatus*, (G): *Triturus marmoratus*, (H): *Lissotriton helveticus*. This number of visits was obtained using the classical equation (see e.g., Pellet and Schmidt 2005).
