# Supplementary figures and images for: Optimizing occupancy surveys by maximizing detection probability: application to amphibian monitoring in the Mediterranean region
Source: Ecol Evol. 2014 Aug 29;4(18):3538–49. doi: 10.1002/ece3.1207 (PMC4224529; doi:10.1002/ece3.1207)

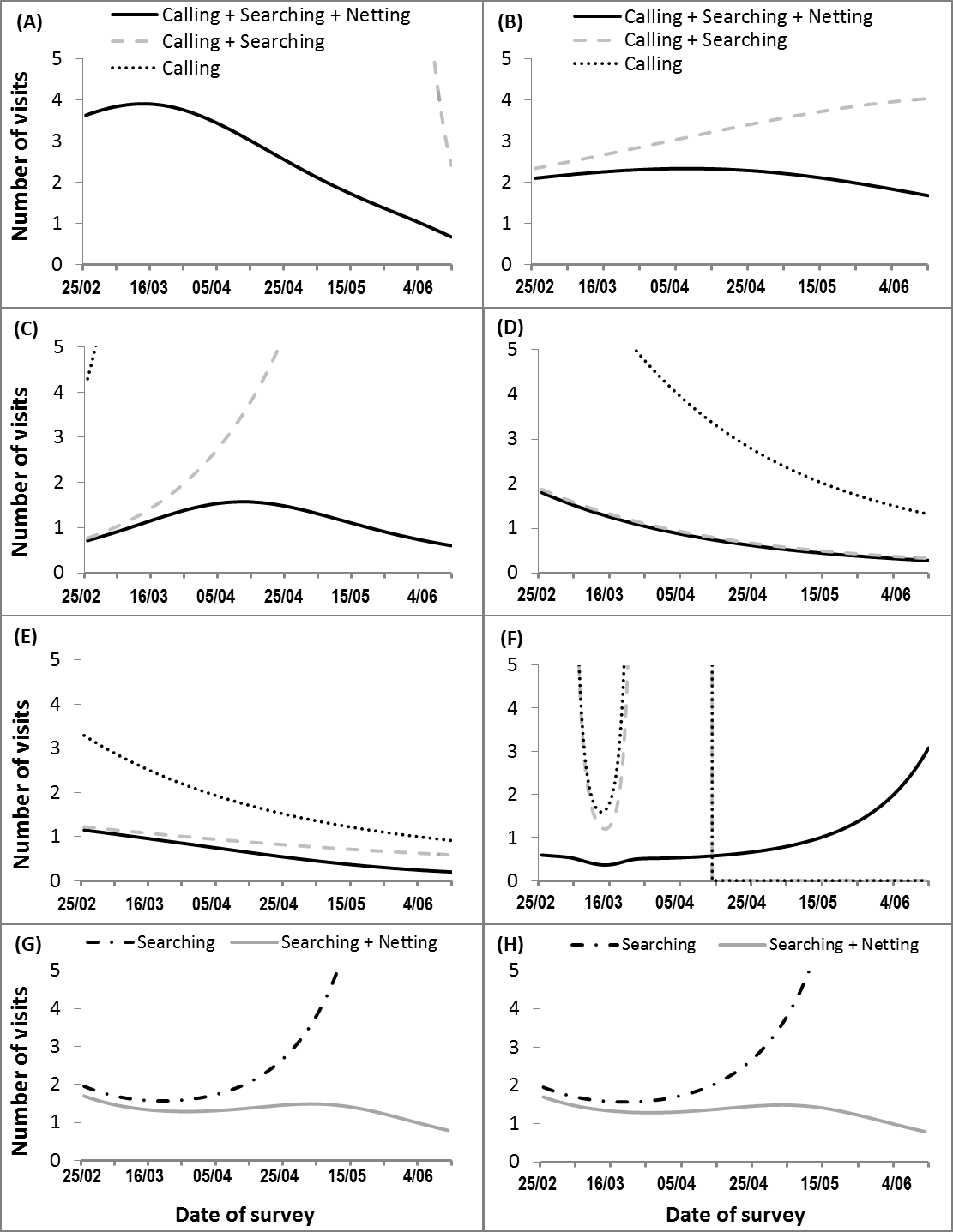

Supplement: Appendix S1 — Number of visits required to detect amphibian species by using one, two, or three methods of detection estimated by using occupancy modeling approach on the 8 species of amphibians detected in 49 ponds of southern France. (A): Alytes obstetricans, (B): Bufo calamita, (C): Bufo bufo, (D): Pelophylax sp., (E): Hyla meridionalis, (F): Pelodytes punctatus, (G): Triturus marmoratus, (H): Lissotriton helveticus. This number of visits was obtained using the classical equation (see e.g., Pellet and Schmidt 2005). [file ece30004-3538-sd1.tif]
